# A Classification Method for Electronic Components Based on Siamese Network

**DOI:** 10.3390/s22176478

**Published:** 2022-08-28

**Authors:** Yahui Cheng, Aimin Wang, Long Wu

**Affiliations:** School of Mechanical Engineering, Beijing Institute of Technology, Beijing 100081, China

**Keywords:** electronic components classification, Siamese network, few-shot learning, vgg, channel correlation loss

## Abstract

In the field of electronics manufacturing, electronic component classification facilitates the management and recycling of the functional and valuable electronic components in electronic waste. Current electronic component classification methods are mainly based on deep learning, which requires a large number of samples to train the model. Owing to the wide variety of electronic components, collecting datasets is a time-consuming and laborious process. This study proposed a Siamese network-based classification method to solve the electronic component classification problem for a few samples. First, an improved visual geometry group 16 (VGG-16) model was proposed as the feature extraction part of the Siamese neural network to improve the recognition performance of the model under small samples. Then, a novel channel correlation loss function that allows the model to learn the correlation between different channels in the feature map was designed to further improve the generalization performance of the model. Finally, the nearest neighbor algorithm was used to complete the classification work. The experimental results show that the proposed method can achieve high classification accuracy under small sample conditions and is robust for electronic components with similar appearances. This improves the classification quality of electronic components and reduces the training sample collection cost.

## 1. Introduction

Electronic components are important parts of electronic devices that are significant to signal amplification, power transmission, and Boolean operations. They are widely used in smartphones, laptops, and other electronic products owing to their excellent performance. However, with the advancement of technology, the research and development cycle of electronic products has become shorter, resulting in a faster iteration of electronic products; thus, many obsolete electronic products become electronic waste. These electronic wastes have many electronic components that are still intact. These electronic components contain materials valuable for producing electronic products [1,2]. Therefore, electronic component classification in these wastes can be better managed for recycling. Current electronic component classification methods mainly rely on experienced staff. In practice, however, there are many different categories of electronic components. Some modern capacitors and resistors are small-surface-mounted, and they look very similar, making it difficult for even experienced staff to classify similar components. This is not only timeconsuming but also laborious, and it is also easy to misclassify electronic components with a similar appearance. At the same time, this requires a high level of skill. Therefore, a new classification method needs to be developed to reduce the probability of classification errors and improve work efficiency.

With the development of deep learning, image classification techniques have made great progress owing to the excellent performance of convolutional neural networks (CNNs). They have become the primary method for solving image classification problems and have been widely applied in many fields [3,4]. Li et al. proposed a new lightweight RegNet model to address the identification of apple leaf diseases rapidly and accurately [5]. Masci et al. proposed a Max-pooling CNN for the classification of steel defects [6]. Zhang et al. presented a new classification method to deal with the breast cancer classification problem by combining a graph neural network (GCN) and convolutional neural network (CNN) [7]. Cai et al. used a novel resolution-aware model for the fine-grained classification of low-resolution images [8]. Wu et al. proposed a novel cross-channel reconstruction network (CCR-Net) for remote sensing image classification [9]. Mou et al. proposed a semisupervised nonlocal graph convolutional network(GCN) for hyperspectral image (HSI) classification [10].

In recent years, several researchers have focused on solving the problem of electronic component classification in the field of electronics manufacturing. Lefkaditis et al. presented a classification algorithm for electronic components which combines support vector machines (SVMs) and multilayer perceptrons (MLPs) with 92.3% accuracy [11]. Atik used CNNs to classify electronic components into three categories (capacitors, diodes, and resistors) to reduce labor intensity and improve efficiency, with a classification accuracy of 98.99% [12]. Salvador et al. combined transfer learning and deep CNNs to classify discrete and surface mounts on electronic prototypes with 94.64% accuracy [13]. Wang et al. proposed a feature selection method using artificial neural networks (ANNs) and two-dimensional patterns of electronic components to classify efficiently and accurately different package types, achieving 95.8% classification accuracy [14]. Conventional electronic component image classification methods cannot effectively combine multiple deep learning features and have poor classifier performance. Their parameters are difficult to optimize, and network convergence is slow. Hu et al. introduced a hierarchical CNN to classify different electronic components with 94.26% accuracy [15]. Although deep learning-based electronic component classification algorithms have achieved better performance, these studies still have some limitations. On the one hand, training neural networks requires a large number of samples to obtain better generalization performance. This process of collecting samples is expensive because of the wide variety of electronic components. If only a few samples are used for training, then the model is prone to overfitting, which leads to the degradation of the recognition performance of the model. On the other hand, to be able to recycle electronic components in e-waste more efficiently, a fine-grained classification of different types of electronic components is required; however, these studies only carried out coarse-grained classification of electronic components. A Siamese network is a symmetric neural network that measures the similarity of a pair of images and is widely applied to solve small sample learning problems [16]. The Siamese network was first introduced by Bromley et al. to solve the signature verification problem [17]. Chopra et al. used convolutional neural networks as the feature extraction part of the Siamese network to address the face verification problem [18]. Some variants have also been developed based on Siamese networks [19,20]. To achieve high classification accuracy with a small number of samples, this study proposed a classification method for electronic components based on the Siamese network, which was used to address the problem that current electronic component classification methods are not applicable to small sample learning. The main contributions of this study are as follows:

(1) The VGG-16 network was selected for the feature extraction part of the Siamese network. The shallow feature maps containing more fine-grained information were fused with the higher-level feature maps containing more semantic information in this model to boost the recognition performance of the model considering limited samples.

(2) To improve further the generalization performance of the model, a channelcorrelation loss function was designed to assist the model in learning the correlation between different channels in the feature map.

The remainder of this paper is organized as follows: Section 2 describes the Siamese network architecture and the datasets used in this study; Section 3 describes the proposed methodology; Section 4 presents the experimental setup, results, and analysis; finally, Section 5 summarizes the conclusions of this study.

## 2. Siamese Network Architecture and Dataset

### 2.1. Siamese Network

**Figure 1:**
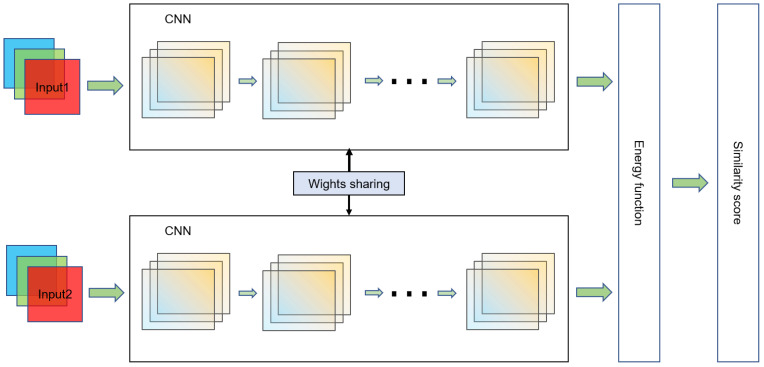
Siamese network architecture diagram.

The Siamese network is used to solve few-shot learning [17,18,21,22]. Figure 1 shows the Siamese network architecture. This network receives a pair of images. Subsequently, two identical feature-extraction subnetworks separately map the pair into a high-dimensional feature space and correlate them using an energy function to determine the similarity of the two images. The two feature-extraction subnetworks share weights, ensuring that the two objects are similarly distant in the high-dimensional feature space. The energy function at the top was used to measure the distance metric between the outputs of each subnetwork.

### 2.2. Dataset

**Figure 2:**
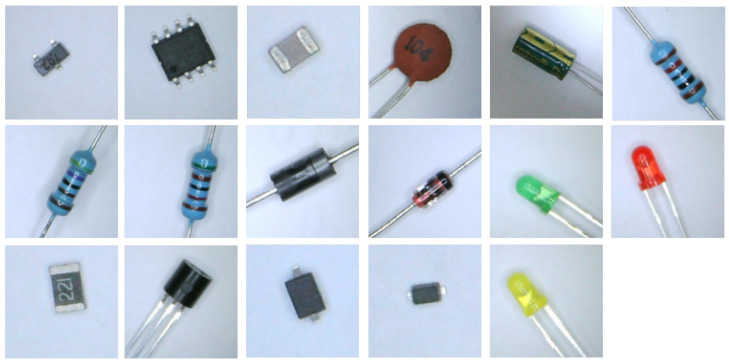
Different kinds of electronic components.

Seventeen different electronic components were captured using a camera to verify the effectiveness of the method proposed in this study, as shown in Figure 2. For simplicity, $n\in \left[1,2,\ldots ,17\right]$ was used to label the different types of electronic components from left to right and top to bottom. Each electronic component contains 182 images. The orientation and position of the electronic component in each image is different. During training, 2/5/10/15 images were randomly selected from each category to train the model. The remaining unselected images were used as the test set to evaluate the model’s performance. It is worth noting that each training sample in the training process consisted of two images from the same category or two images from different categories.

## 3. Method

### 3.1. Improved VGG-16 Model

**Figure 3:**
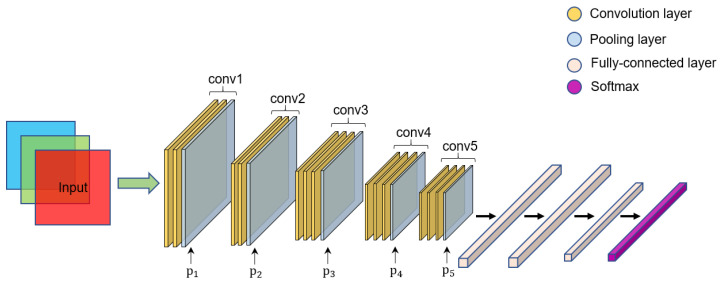
VGG-16 model architecture.

Figure 3 shows the VGG-16 [23] CNN architecture. A subnetwork of five convolutional blocks first extracts the image for feature extraction, and the extracted features are fed into three subsequent fully connected layers for classification. Each convolutional block consists of convolutional layers, activation functions, and a maximum pooling layer. The first two convolutional blocks contain two convolutional layers, and the last three convolutional blocks contain three convolutional layers. All convolutional layers use a 3 × 3 convolutional kernel. The activation function is a rectified linear unit (ReLU) [24], and the feature map is halved in height and width after passing through the maximum pooling layer. A dropout [25] with 0.5 probability is used to mitigate model overfitting in the first three fully connected layers after the fifth convolutional block. Finally, the classification result is obtained by the softmax operation.

**Figure 4:**
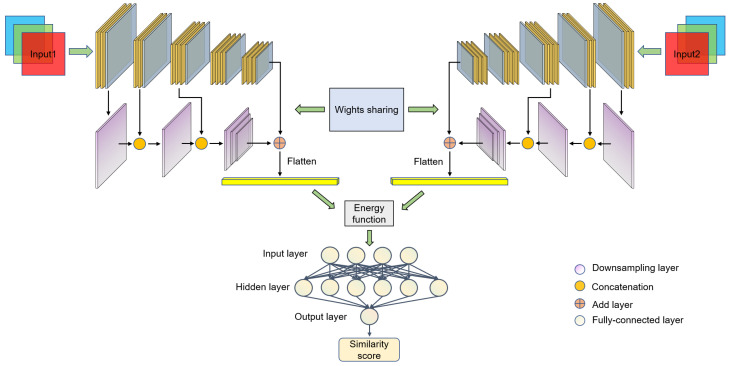
Proposed Siamese network model architecture.

In order to improve the discriminative performance of the model under small sample conditions, the improved VGG-16 model was proposed and denoted as VGG-16-F. Figure 4 shows the classification model using VGG-16-F as the feature extraction part of the Siamese network. In practical applications, the model complexity is usually considered, which is measured by floating-point operations (FLOPs) and the number of parameters. Therefore, the last three fully connected layers in VGG-16 are deprecated to reduce the computational overhead and model complexity. In addition, considering that the feature map obtained from the low-level convolutional layer has more fine-grained information about the object, the feature map obtained from the conv1_1 convolutional layer was downsampled by a factor of two and concatenated with the feature map obtained from the conv2_1 convolutional layer in the channel dimension. The same operation was performed on the result and feature map generated by the conv3_1 layer. Finally, after passing through three consecutive downsampling layers, a fusion operation with the feature map from $p_{5}$ is performed to obtain *F*. The downsampling layer adopts a 3 × 3 convolutional kernel and stride size of 2. The distance between the flattened F and output of the other branch of the Siamese network is calculated. Specifically, Equation (1) is used as the energy function.

(1) $$F_{o}=\left|F_{1}-F_{2}\right|$$

where $F_{1}$ and $F_{2}$ denote the feature vector from different branches of the Siamese network, respectively, and $F_{o}$ denotes the output feature vector after the element-by-element difference and absolute value operations are performed on $F_{1}$ and $F_{2}$. Subsequently, the similarity S between the two is obtained after passing through two fully connected layers whose hidden units are 256 and 1, respectively. The dropout rate was set to 0.1, and the activation function was sigmoid. S is 1 if the two images are from the same class; otherwise, S is 0.

### 3.2. Loss Function

The loss function is used as the optimization objective of the training model, which is minimized to reduce the gap between the predicted value and label. Koch et al. [26] employed cross-entropy loss with $L_{2}$ regularization to train a Siamese network. Crossentropy loss is defined as follows:

(2) $$L_{v}=-Llogp-\left(1-L\right)log\left(1-p\right)$$

where *p* denotes the output of the Siamese network, and *L* is the label of the two images $I_{1}$ and $I_{2}$ fed into the Siamese network. *L* is 1 if the images are from the same class; otherwise, it is 0.

In the field of image style transfer, Gatys et al. employed a feature space consisting of correlations between different filter responses to obtain a style representation of an input image and reduce the difference in style between the generated image and this input image by minimizing the style loss [27]. Inspired by their work, the channel correlation loss $L_{c}$ that allows the model to learn channel-to-channel correlation was designed and optimized together with $L_{v}$ to further improve the generalization performance of the model with limited samples. For images from different classes, the correlation between different channels in their respective output feature maps is distinct; therefore, it is beneficial to allow the neural network to learn about the correlation between them. The feature map of the *m*th layer is denoted as $F_{m}\in R^{c_{m}\times h_{m}\times w_{m}}$, where $c_{m}$, $h_{m}$ and $w_{m}$ refer to the number of channels, height, and width of the layers, respectively. Subsequently, the feature map on each channel is expanded into a vector, thus obtaining a matrix $F_{m}^{\prime }$ of size $c_{m}\times s_{m}$, that is,

(3) $$F_{m}^{\prime }=\left[\begin{array}{c} f_{1}\\ f_{2}\\ \vdots \\ f_{c_{m}} \end{array}\right]$$

where $s_{m}=h_{m}\times w_{m}$. Thereafter, matrix $Q_{m}\in R^{c_{m}\times s_{m}}$ is obtained by normalizing $f_{i}$, $i\in \left[1,2,\ldots ,c_{m}\right]$ according to Equation (4).

(4) $$q_{i}=\frac{f_{i}}{∥f_{i}{∥}_{2}+\varepsilon }$$

where ε is a small positive constant used to maintain numerical stability, which is set to $10^{-8}$ in the experiments. Based on the above discussion, the Gram matrix $G^{m}=Q_{m}Q_{m}^{T}\in R^{c_{m}\times c_{m}}$ for the *m*th layer is obtained. Gram matrix of $I_{1}$ is denoted as $G^{1m}$ and that of $I_{2}$ as $G^{2m}$. From this, we can obtain the similarity between the two as follows:

(5) $$D_{m}=\sum_{i}\sum_{j}W_{ij}^{m}⊙\left(G_{ij}^{1m}-G_{ij}^{2m}\right)⊙\left(G_{ij}^{1m}-G_{ij}^{2m}\right)$$

where $G_{ij}^{m}$ is located in the *i*th row and *j*th column of the Gram matrix; and $W_{ij}^{m}$ is located in the *i*th row and *j*th column of the weight matrix $W^{m}$. This weight matrix is obtained by softmax operation on each row, which is defined as

(6) $$W^{m}=softmax\left(G^{1m}\right)$$

The channel correlation loss function can then be defined as

(7) $$L_{c}=\sum_{m}LD_{m}+\left(1-L\right)e^{-D_{m}}$$

where m∈{conv1_1,conv2_1,conv3_1,conv4_1,conv5_1}.

Therefore, the total loss is written as

(8) $$L=L_{v}+L_{c}$$

During training, the cross-entropy loss $L_{v}$ and channel correlation loss $L_{c}$ are minimized to allow the Siamese network to learn to discriminate whether two images are input to the same class.

## 4. Experiment Results and Discussion

### 4.1. Experimental Details

In this study, all experiments were conducted under the same conditions. The graphics processing unit adopted was GeForce GTX 1060 (6 GB). The model was built using the PyTorch deep learning framework. The total number of training epochs was 120, and the Adam optimizer [28] was used to update the model’s parameters, where $\beta_{1}$ = 0.9, and $\beta_{2}$ = 0.999. The batch size was four. The initial learning rate lr was set to 0.0001. The learning rate was dynamically adjusted in each epoch T according to $lr^{T+1}=0.92lr^{T}$. The size of the image used in the training model was 112 × 112. For the convolutional layer, the weights were initialized using He initialization [29] and the biases were initialized to 0. For the fully connected network, the weights were initialized using a normal distribution with a mean of 0 and a standard deviation of 0.01, and the biases were also initialized to 0. In the training, the images were horizontally flipped and angularly rotated with a probability of 0.5, where the angle varied in the range [$-5^{\circ }$, $5^{\circ }$].

Since the Siamese neural network outputs the similarity of a pair of input images rather than the category to which the electronic components in the images belong, after the training was completed, we used the nearest neighbor algorithm to classify the images. Specifically, we first collected one sample from each category of electronic components and classified these samples into a new dataset. Then, we compared the test images with the images of each category in this dataset; the more similar the two images were, the higher the prediction score output by the Siamese neural network, and vice versa. Finally, we selected the category with the highest score as the classification result.

In our experiments, we performed each set of experiments three times, and in each training we selected the model parameters with the best discriminative performance in the last 10 epochs for classification tests, and finally the model parameters with the best classification performance in these three results were selected.

### 4.2. Evaluation Metrics

In this study, the classification performance of the model was evaluated using an accuracy rate defined as follows:

(9) $$accuracy=\frac{C}{T}$$

where *C* denotes the number of correct classifications, and *T* denotes the total number of classifications performed. Additionally, the area under the receiver operating characteristic curve (AUC) was used to measure the discriminative performance of the model. The horizontal coordinate of the receiver operating characteristic (ROC) curve is the false positive rate (FPR), and the vertical coordinate is the true positive rate (TPR), both of which are defined as follows:

(10) $$FPR=\frac{FP}{FP+TN}$$

(11) $$TPR=\frac{TP}{TP+FN}$$

where false positive (FP) denotes that the model predicts a positive result for a sample, but the true label is negative; true negative (TN) indicates that the prediction results and labels of the sample are both negative; true positive (TP) means that the prediction results and labels of the sample are both positive; and false negative (FN) refers to a sample with a negative prediction result and a positive label.

### 4.3. Experimental Results

To verify the effectiveness of the method proposed in this study, the classification accuracy was tested when using VGG-16, VGG-16-F based on feature fusion, VGG-16 with channel correlation loss, and VGG-16 with both methods included as feature extraction subnets of the Siamese network for different sample numbers. Table 1 summarizes the results of the ablation experiments.

Table 1 shows that the accuracy of different models presents an increasing trend as the number of training samples increases. This indicates that the larger the number of samples, the better the neural network distinguishes between different classes of objects. The classification accuracy of the VGG-16-F model was improved by 0.15, 0.11, 0.10, and 0.15, respectively, compared to the VGG-16 model for training sample sizes of 2, 5, 10, and 15, indicating that propagating the fine-grained information contained in the lower convolutional layers to the higher convolutional layers helps to improve the discriminative performance of the model. The accuracy of the VGG-16 model using channel correlation loss compared to the baseline improved by 0.07, 0.06, 0.08, and 0.13, respectively, suggesting that channel correlation loss helps the neural network learn the relationship between different channels in the feature map, thus improving the ability to discriminate different classes of objects. Furthermore, the accuracy of VGG-16-F combined with channel correlation loss improved by 0.16, 0.15, 0.24, and 0.23, respectively, compared with the original VGG-16, which indicates that the combination of the two methods can improve the classification accuracy of the model.

**Figure 5:**
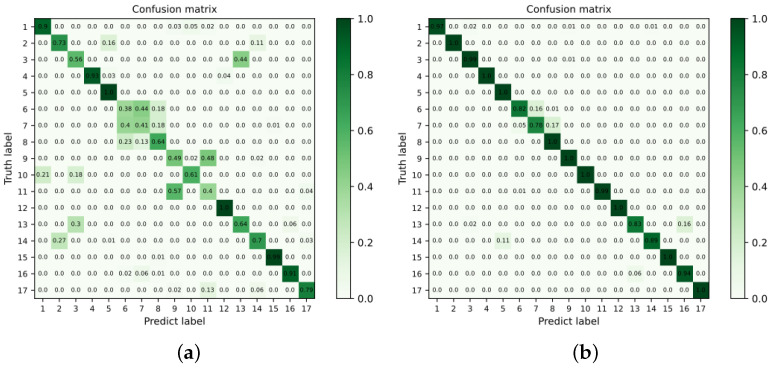
Confusion matrix of testing results. (**a**) VGG-16. (**b**) VGG-16-F&$L_{c}$.

Figure 5 presents the confusion matrix of the VGG-16 model and the improved model under the condition of 15 training samples, whose values on the diagonal indicate the probability of correct classification. As shown in Figure 5a, the original model can accurately classify electronic components with significantly different appearances. However, it is prone to the misclassification of electronic components with similar appearances. As shown in Figure 5b, the values on the diagonal are above 0.75, indicating that the model proposed in this study can classify each electronic component more accurately. It is a significant improvement relative to the baseline, further demonstrating the effectiveness of the method proposed in this study.

Moreover, experiments with AlexNet [30], ResNet-34, ResNet-50 [31], and GoogleNet [32] were also performed as feature extraction subnetworks in the Siamese network; the experimental results are shown in Table 2.

The ResNet-50 accuracy was lower than that of ResNet-34 because the former has more parameters than the latter and is prone to overfitting. Given the same number of training samples, the accuracy of GoogLeNet was higher than that of the other three networks because GooLeNet uses a stacked inception block. It extracts features from images at different scales and reduces model complexity, but its accuracy is still lower than that of the proposed method.

**Figure 6:**
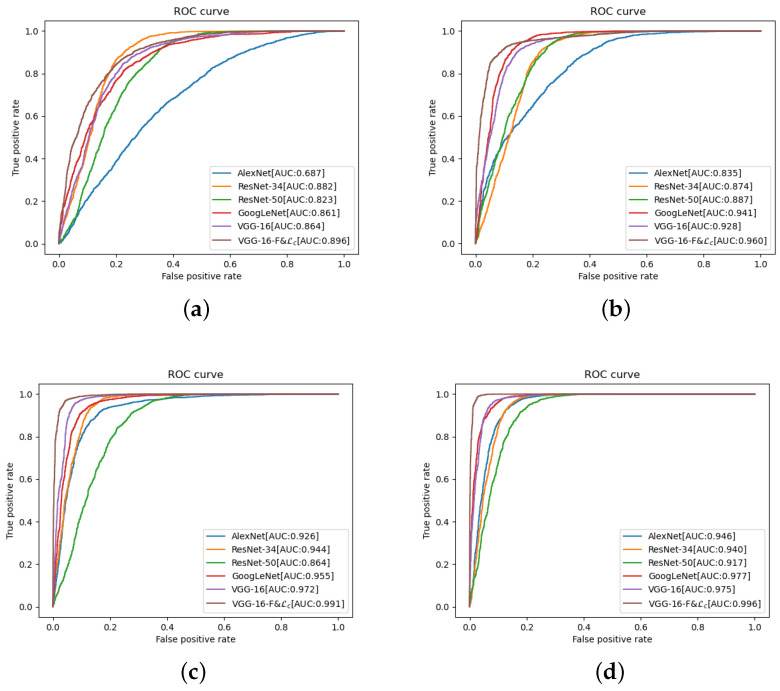
ROC curves of different models. (**a**) Number of training samples: 2. (**b**) Number of training samples: 5. (**c**) Number of training samples: 10. (**d**) Number of training samples: 15.

Figure 6 shows the ROC curves of the different models for different numbers of training samples to evaluate the discriminative performance of the method proposed in this study for a pair of images. The figure shows that the AUC of the proposed method is higher than that of the other models for different numbers of training samples, indicating that its discriminative performance is better than that of the other models.

Therefore, combining the improved VGG-16 model with channel correlation loss improved the model’s classification accuracy while outperforming other outstanding classification networks.

## 5. Conclusions

Electronic component classification in electronic waste facilitates better recycling. Current deep learning-based classification algorithms require many training samples to obtain high performance and do not provide a fine-grained classification level. This study proposed a Siamese network-based classification algorithm for electronic component classification with high accuracy under small sample constraints to address these issues. First, the low-level feature map of the VGG-16 model was extracted. A continuous downsampling operation was performed to improve the discriminative performance of the Siamese network by fusing the features with the high-level feature map. Second, a channel correlation loss function that allows the model to learn correlation information about different channels in the feature map was proposed to boost the model’s discriminative ability further. For inference, the nearest neighbor algorithm was used for electronic component classification. The experimental results show that the proposed method can achieve 0.94 accuracy with 15 training samples. This is higher than that of other CNNs used as the feature extraction part of the Siamese network, such as AlexNet, ResNet-34, ResNet-50, and GoogLeNet. The results confirm the effectiveness of the proposed method. Although this approach achieves better results, the method still suffers from classification errors for electronic components with similar appearances. In future work, we will apply the attention mechanism to our model to improve the robustness for similar electronic components and investigate the effect of the model on electronic component recognition under different backgrounds and image qualities. Furthermore, our method will be further applied to more types of electronic components and other small sample classification tasks.

## Figures and Tables

**Table 1:** Results of ablation experiments.

Model	$N^{1}=2$	N=5	N=10	N=15
VGG-16	0.31	0.50	0.67	0.71
VGG-16-F	0.46	0.61	0.77	0.86
VGG-16&$L_{c}$	0.38	0.56	0.75	0.84
VGG-16-F&$L_{c}$	0.47	0.65	0.91	0.94

^1^ The number of training samples for each type of electronic component.

**Table 2:** Comparison of experimental results with other networks.

Model	$N^{1}=2$	N=5	N=10	N=15
AlexNet	0.15	0.34	0.49	0.55
ResNet-34	0.31	0.39	0.44	0.47
ResNet-50	0.18	0.29	0.31	0.36
GoogLeNet	0.37	0.52	0.58	0.78

^1^ The number of training samples for each type of electronic component.

## Data Availability

Not applicable.
